# Design and Characterization of Sustainable PLA-Based Systems Modified with a Rosin-Derived Resin: Structure–Property Relationships and Functional Performance

**DOI:** 10.3390/biomimetics10120801

**Published:** 2025-12-01

**Authors:** Harrison de la Rosa-Ramírez, Miguel Aldas, Cristina Pavon, Franco Dominici, Marco Rallini, Debora Puglia, Luigi Torre, Juan López-Martínez, María Dolores Samper

**Affiliations:** 1 Instituto de Tecnología de Materiales (ITM), Universitat Politècnica de València (UPV), Plaza Ferrándiz y Carbonell 1, 03801 Alcoy, Spain; hardela@epsa.upv.es (H.d.l.R.-R.); jlopezm@mcm.upv.es (J.L.-M.); masammad@upv.es (M.D.S.); 2 Departamento de Ciencia de Alimentos y Biotecnología, Facultad de Ingeniería Química y Agroindustria, Escuela Politécnica Nacional, Quito 170517, Ecuador; miguel.aldas@epn.edu.ec; 3 Civil and Environmental Engineering Department, University of Perugia, Strada di Pentima 4, 05100 Terni, Italy; franco.dominici@unipg.it (F.D.); marco.rallini@unipg.it (M.R.); debora.puglia@unipg.it (D.P.); luigi.torre@unipg.it (L.T.)

**Keywords:** poly(lactic acid) (PLA), rosin-derived resin, bio-based polymers, structure–property relationships, thermomechanical behavior, sustainable materials

## Abstract

The design of sustainable polymer systems with tunable properties is essential for next-generation functional materials. This study examines the influence of a phenol-free modified rosin resin (Unik Print™ 3340, UP)—a maleic anhydride- and fumaric acid-modified gum rosin—on the structural, thermal, rheological, and mechanical behavior of four poly(lactic acid) (PLA) grades with different molecular weights and crystallinity. Blends containing 3 phr of UP were prepared by melt compounding. Thermogravimetric analysis showed that the incorporation of UP did not alter the thermal degradation of PLA, confirming stability retention. In contrast, differential scanning calorimetry revealed that UP affected thermal transitions, suppressing crystallization and melting in amorphous PLA grades and shifting the crystallization temperature to lower values in semi-crystalline grades. The degree of crystallinity decreased for low-molecular-weight semi-crystalline PLA but slightly increased in higher-molecular-weight samples. Mechanical tests indicated that UP acted as a physical modifier, increasing toughness by over 25% for all PLA grades and up to 60% in the amorphous, low-molecular-weight grade. Rheological measurements revealed moderate viscosity variations, while FESEM analysis confirmed microstructural features consistent with improved ductility. Overall, UP resin enables fine tuning of the structure–property relationships of PLA without compromising stability, offering a sustainable route for developing bio-based polymer systems with enhanced mechanical performance and potential use in future biomimetic material designs.

## 1. Introduction

Plastics are widely used in modern society, but the increasing consumption and disposal of conventional petrochemical polymers have raised serious environmental concerns due to their persistence and pollution potential [1]. In response, bio-based and biodegradable polymers have gained growing attention as sustainable alternatives. These materials, derived from renewable resources, offer advantages such as reduced fossil fuel dependency, lower production energy requirements, and enhanced recyclability. When properly modified, biopolymers can replace traditional plastics in applications including food packaging, agriculture, and domestic goods.

Among them, poly(lactic acid) (PLA) stands out as one of the most extensively studied and commercially relevant bio-based polymers due to its biodegradability, favorable mechanical properties, and processability [2,3,4]. PLA is produced via polymerization of lactic acid, where parameters such as temperature, pressure, and pH, as well as the D/L-lactide ratio, determine the molecular weight and crystalline structure of the resulting polymer [5]. These structural variations lead to a wide range of PLA grades, from amorphous to semi-crystalline, influencing their thermomechanical and rheological behavior. Selecting the appropriate PLA type for specific applications is therefore crucial, particularly when developing modified formulations with enhanced performance. Recent studies have further highlighted how rheological tailoring—through thermal or photo-induced modification—can extend the processing window and mechanical tunability of PLA [6,7].

Gum rosin is a natural resin obtained from pines and conifers, valued for its availability, low cost, and biodegradability. Chemically, rosin consists mainly of resin acids and neutral diterpenoid compounds [8,9]. Traditional chemical modifications often involve phenol or formaldehyde, but environmental and health concerns have encouraged the development of phenol-free alternatives. Modified rosin derivatives prepared with acrylic or maleic acids have demonstrated good performance in coatings, adhesives, and polymeric blends, highlighting their versatility as sustainable additives [8,10]. Recent work (2024) continues to expand this field, demonstrating how bio-based natural resins and esters can reinforce polymer networks and promote partial miscibility without solvents [11].

Several recent studies have investigated rosin-based and other bio-sourced additives for tailoring the performance of poly(lactic acid) (PLA) and related biopolymers. Pavon et al. [12] developed bilayer films using poly(ε-caprolactone) coated with electrosprayed gum rosin microspheres, while Aldas et al. [13] used rosin derivatives to improve miscibility and processability in thermoplastic starch systems. Similarly, previous work has shown that rosin esters can act as lubricants in PLA, promoting improved flow and processability [12,14]. More recent studies (2023–2025) have demonstrated how modified rosin derivatives and bio-based plasticizers influence crystallization, ductility, and processability in PLA systems [15,16]. In addition, Plamadiala et al. [17] reviewed the influence of various natural and synthetic additives on PLA mechanical and rheological performance in fused deposition modeling (FDM), highlighting the current trend toward additive-guided tuning of PLA flow and processability. These include investigations on phenol-free rosin resins improving biodegradability and melt stability [16], as well as natural resin esters acting as sustainable compatibilizers in PLA-based blends [14,15]. Such advances align with the current shift toward rheology-guided design and circular processing of PLA composite [18].

Together, these reports confirm the potential of rosin derivatives as functional additives for sustainable polymer design, although most have focused on a single PLA grade or specific formulation. In contrast, the present study systematically investigates a phenol-free modified rosin resin (Unik Print™ 3340, UP) at a fixed loading of 3 phr across four PLA grades differing in molecular weight and crystallinity, thereby establishing new structure–property relationships linking thermal, rheological, and mechanical behavior. Specifically, this work designs and characterizes sustainable PLA–UP formulations to evaluate how the incorporation of this maleic-anhydride- and fumaric-acid-modified gum rosin influences the performance of various PLA matrices. The selected concentration of 3 phr, previously identified as optimal and below the saturation threshold, ensures homogeneous dispersion and stable mechanical and thermal behavior [16]. The resulting materials were characterized by infrared spectroscopy, scanning electron microscopy, thermogravimetric analysis, differential scanning calorimetry, and mechanical testing. This approach provides new insights into how molecular structure governs the thermal, rheological, and mechanical responses of PLA after UP incorporation. While previous studies have mainly explored rosin derivatives as plasticizers or compatibilizers, few have systematically examined their effects across different PLA grades. The findings contribute to understanding how natural resin derivatives can tailor PLA performance, offering a sustainable route for developing bio-based polymer systems with tunable thermomechanical behavior and improved functional properties.

## 2. Materials and Methods

### 2.1. Materials

Four commercial grades of biodegradable poly(lactic acid) (PLA) with number-average molecular weights ranging from 59 to 245 kg·mol^−1^ [19,20,21,22] were used as polymeric matrices. Two Luminy^®^ grades (LX-175 and L130) were supplied by Corbion Purac (Amsterdam, The Netherlands), and two Ingeo™ grades (2003D and 6201D) were obtained from NatureWorks LLC (Minnetonka, MN, USA).

A complex phenol-free modified rosin resin, Unik Print™ 3340 (UP), supplied by United Resins (Figueira da Foz, Portugal), was used as the bio-based additive. UP is a maleic anhydride- and fumaric acid-modified rosin with a softening point of 135 °C, an acid value below 35 mg KOH·g^−1^, and a viscosity of 15–30 Pa·s (measured at 23 °C and 25 s^−1^).

The main physical properties and molecular characteristics of the selected PLA grades are presented in Table 1. The information was obtained from suppliers’ technical data sheets and literature.

### 2.2. Blends Compounding and Sample Preparation

Binary PLA–UP blends containing 3 phr of UP resin were prepared by melt compounding, following the conditions described in a previous work [16]. The compositions and corresponding sample labels are summarized in Table 2. A concentration of 3 phr was selected based on our previous optimization study [16], which identified this value as the maximum below the saturation threshold that ensures homogeneous dispersion and reproducible mechanical and thermal behavior in PLA matrices.

Prior to processing, all materials were dried at 50 °C for 24 h. The PLA pellets and UP resin were manually premixed and then compounded using a twin-screw extruder (Haake Rheocord, Thermo Fisher Scientific, Karlsruhe, Germany) with an L/D ratio of 24:1. The temperature profile was set from die to hopper as 180/180/175/170/160/60 °C, with a screw speed of 20 rpm.

The resulting blends were pelletized and subsequently processed by injection molding using a Sprinter 11t (Erinca S.L., Barcelona, Spain). Molding was carried out at 175–185 °C, with an injection time of 2 s and a cooling time of 40 s. Standard test specimens were produced according to ISO 527-2 [23] for tensile testing and ISO 179-1 [24] for impact testing.

### 2.3. Thermal Characterization of PLA Blends with UP Resin

The thermal stability of the processed samples was evaluated via thermogravimetric analysis (Seiko Exstar 6300, Tokyo, Japan). Weight changes of samples (13–15 mg), placed in alumina crucibles, were recorded during a dynamic heating cycle from 30 °C to 700 °C at a constant heating rate of 10 °C·min^−1^ in a nitrogen atmosphere (30 mL·min^−1^). The onset degradation temperatures (*T_5%_*) of the samples were determined at a 5% loss of their initial mass, whereas the temperatures of the maximum degradation rate (*T_max_*) were determined from the corresponding peak of the first derivative of the TGA curves (DTG).

DSC tests were conducted in a Q200 calorimeter from TA Instruments (New Castle, DE, USA). 8 mg average weight samples sealed in standard aluminum pans of 40 µL were subjected to three cycles of dynamic thermal analysis program: (1) heating cycle from 30 °C to 190 °C, (2) a cooling cycle from 190 °C to −30 °C, and (3) a second heating cycle from −30 °C to 200 °C. Tests were conducted at a heating/cooling rate of 10 °C·min^−1^ under an inert N_2_ atmosphere (30 mL·min^−1^). The crystallinity degree (*X_c_*) of different PLA grades and PLA blends was reported and calculated using Equation (1):

(1) $$X_{C}=\;\left[\frac{∆H_{m}-∆H_{cc}}{∆H_{m}^{0}\cdot (1-w)}\times 100\;\right],$$

where ∆*H_m_* is the thermodynamic melting enthalpy (Jg^−1^) of each sample taken from the thermal curves of the second heating cycle, ∆*H_cc_* is the cold crystallization enthalpy (Jg^−1^), and ∆*H*^0^_m_ is considered as the theoretical melting enthalpy of a 100% crystalline PLA, i.e., 93.0 (Jg^−1^) [25], and (1 − *w*) corresponds to the weight fraction of PLA in the sample blends.

### 2.4. Fourier Transform Infrared Spectroscopy (FTIR) Analysis

The interaction of the different PLA matrices with the UP resin was examined by Fourier Transform Infrared Spectroscopy Analysis (FTIR) using a JASCO 615 plus spectrometer (Easton, MD, USA). The sample spectra were recorded with 118 consecutive scans at 4 cm^−1^ resolution in the wavelength between 4000 and 600 cm^−1^ overwritten in a background spectrum, previously registered to compensate for the humidity effect and presence of carbon-dioxide in the air.

### 2.5. Mechanical Properties

To evaluate the influence of UP resin incorporation on the mechanical properties of the different PLA grades as a function of their molecular weight, tensile tests were performed using a universal electronic tensile-tester ELIB 30 from S-A-E.-Iberstest (Madrid, Spain) by setting a cross-head speed of 10 mm·min^−1^, with a load cell of 5 kN according to ISO 527-2 guidelines [23]. Tests were conducted in five standard testing specimens (dumbbell-shape “1BA”) obtained by injection-molding. The stress–strain curves, obtained in the uniaxial tensile tests, are reported as test results. In addition, the toughness values calculated from the area under the stress–strain curves are reported.

### 2.6. Rotational Rheology

The rheological characterization of different PLA grades and their corresponding formulations with UP resin was obtained by using an Ares N2 rheometer from Rheometric Scientific (Reichelsheim, Germany), with parallel plates geometry (25 mm diameter) at 1.5 mm gap. The dynamic temperature ramp was performed by heating from 160 °C to 250 °C at a rate of 3 °C·min^−1^, a frequency of 1 Hz and a maximum strain (γ) maintained at 3%, which was previously verified to be in the linear regime of the viscoelastic response of the materials. The verification of the linear regime was performed by a dynamic strain sweep test, performed at 185 °C covering the strain range of 0.5–8% with a strain increment of 0.5%. The complex viscosity (η*) as a function of the temperature is reported.

### 2.7. Morphological Evaluation

The morphology of the cross-section surfaces of the different PLA grades and their corresponding formulations with UP resin was observed and characterized by Field Emission-Scanning Electron Microscope (FESEM, ZEISS SUPRA 25, Carl Zeiss Microscopy GmbH, Oberkochen, Germany) operated at 2 kV. Prior to observation, all samples were gold-coated using an automatic sputter coater (Agar-B7341, Agar Scientific Ltd., Stansted, UK) to improve surface conductivity.

## 3. Results and Discussion

### 3.1. Thermal Behavior of TPS/PVA Materials

**Figure 1:**
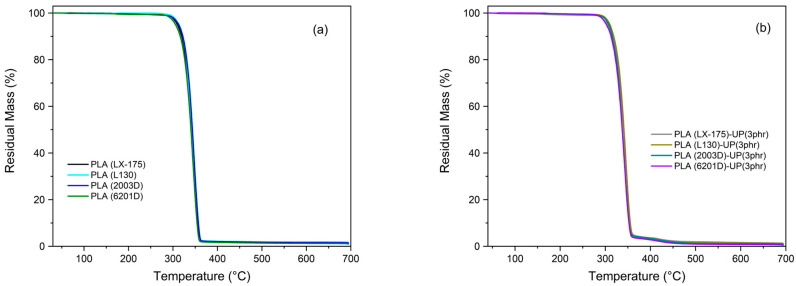
Thermogravimetric (TGA) curves of the tested samples: (**a**) neat PLA grades; (**b**) PLA grades modified with 3 phr of UP.

The thermal degradation of PLA is primarily attributed to the susceptibility of its ester linkages to temperature, which promotes random backbone scission reactions [26,27]. When analyzing the thermal degradation behavior of the different PLA grades, no significant variation in the decomposition profiles was observed (Figure 1) consistent with the results of Atalay et al. [28], who reported that PLA degradation is independent of both the D/L enantiomer ratio and molar mass.

**Figure 2:**
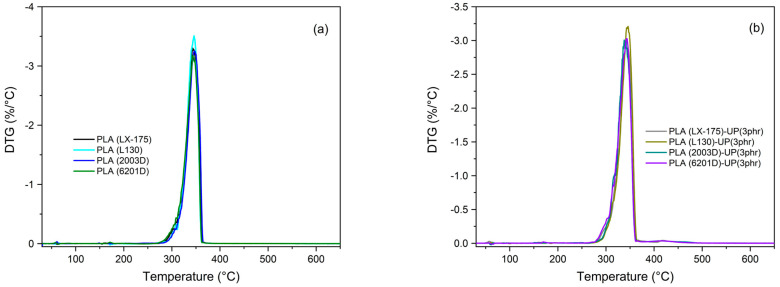
Derivative thermogravimetric (DTG) curves of the PLA samples: (**a**) neat PLA grades; (**b**) PLA grades modified with 3 phr of UP. The thermal transitions of PLA depend strongly on the D/L-lactide ratio, which determines chain regularity and crystallization potential [30]. Figure 2a shows that the amorphous PLAs (LX-175 and 2003D) exhibited broad exothermic peaks between 110–145 °C and 120–140 °C, respectively, corresponding to cold crystallization. Their weak melting peaks confirm the limited ability to form crystalline domains due to their high D-lactic acid content [31]. In contrast, the semi-crystalline PLA grades (L130 and 6201D) displayed clear cold-crystallization peaks (90–110 °C and 105–125 °C, respectively), followed by distinct melting endotherms, reflecting the dominance of L-lactide sequences that favor crystal formation.

After incorporating 3 phr of the modified rosin resin (UP), the thermal degradation behavior of all PLA grades remained practically unchanged. The UP resin did not affect degradation kinetics or stability, as shown in Figure 1b and Figure 2b. Both the onset degradation temperature (*T_5_%*) and maximum degradation rate temperature (*Tₘₐₓ*) remained nearly constant. The residual mass at 600 °C was also comparable to that of neat PLA samples, indicating that UP did not alter the decomposition pathway. Similar results were reported by Wan et al. [29], or PLA blended with amorphous and crystalline cellulose, where no significant effect on degradation was detected. These observations confirm that UP acts as a physically dispersed component, not as a reactive stabilizer.

Upon incorporation of UP resin (Figure 2b), the amorphous PLA grades showed a strong reduction—or complete disappearance—of both cold-crystallization and melting peaks, particularly for PLA (2003D)-UP (3 phr). In PLA (LX-175)-UP (3 phr), these transitions were barely visible, indicating that UP further restricted molecular mobility and hindered the development of ordered regions.

**Figure 3:**
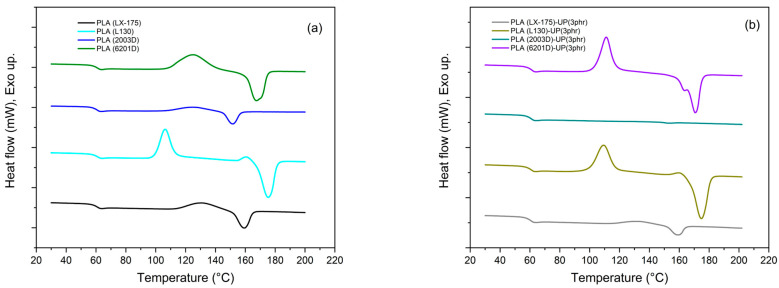
Differential Scanning Calorimetry (DSC) curves of PLA samples: (**a**) neat PLA grades; (**b**) PLA grades modified with 3 phr of UP.

For semi-crystalline PLA grades, the effect of UP was dependent on molecular weight. In PLA (6201D)-UP (3 phr), the cold-crystallization peak shifted from 125 °C to 111 °C, with increased intensity and a small pre-melting shoulder, suggesting a modified crystallization pathway or formation of multiple crystalline domains [32], as shown in Figure 3. In contrast, PLA (L130)-UP (3 phr) exhibited only a slight *Tcc* increase (~3 °C) without changes in melting behavior.

Similar additive-dependent trends have been reported in the literature. Piekarska et al. [32] observed enhanced crystallization in PLA containing calcium carbonate, whereas Perinović et al. [33] found that magnesium hydroxide reduced crystal formation. These contrasting effects highlight the importance of additive chemistry and compatibility in governing PLA crystallization behavior. The bio-based rosin derivative used in this study, being organic and partially compatible, induces distinct molecular interactions compared with inorganic fillers.

The degree of crystallinity (Xc, Table 3), supports these observations. Small melting endotherms detected in PLA (LX-175)-UP and PLA (2003D)-UP correspond to trace crystallinity (<0.5%), which lies within the precision limit of DSC quantification. These values are therefore reported as <0.5% in Table 3. The amorphous PLA grades exhibited negligible crystallinity (Xc < 2%), which was completely suppressed after UP addition. In contrast, the semi-crystalline grades displayed opposite trends: Xc decreased by approximately 80% for PLA (6201D) but increased by about 30% for PLA (L130). The reduction in Xc for 6201D correlates with its enhanced ductility, whereas the increase observed for L130 suggests partial reinforcement of the crystalline phase and improved structural packing.

Overall, the results show that the UP-resin acts as a bio-based molecular modifier capable of tuning the thermal transitions of PLA depending on its molecular architecture. This behavior aligns with the principles of biomimetic material design, where structural organization governs function, demonstrating the potential of natural resin derivatives to modulate crystallization and viscoelastic behavior in sustainable polymer systems.

Although small variations in melting temperature (*Tm*) were observed among the PLA–UP blends, these differences are attributed to changes in chain mobility and nucleation effects induced by the UP resin rather than to chemical modification of PLA. The consistent onset degradation temperatures observed in TGA confirm that the incorporation of UP does not compromise the thermal stability of PLA or induce significant chemical changes in the polymer backbone. These results indicate that UP acts primarily as a physical modifier influencing molecular packing, rather than as a reactive additive.

### 3.2. FTIR Analysis of TPS/PVA Blend

**Figure 4:**
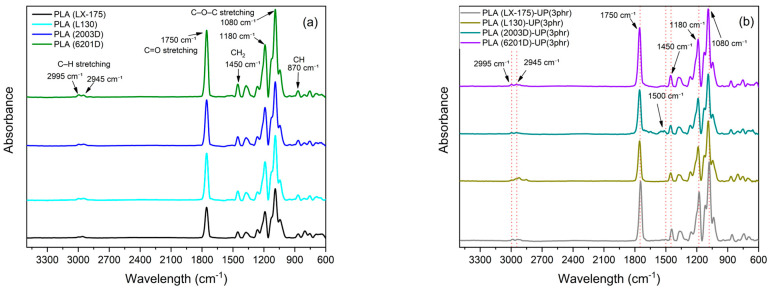
FTIR spectra of the different PLA grades and their corresponding UP-modified formulations.

The FTIR spectra of the different PLA grades and their corresponding formulations with UP resin are presented in Figure 4. All spectra exhibited similar profiles, indicating that the addition of UP resin did not significantly alter the chemical structure of PLA.

Characteristic absorption bands of PLA were observed at approximately 2995–2945 cm^−1^ (asymmetric and symmetric C–H stretching of CH_3_ groups), 1750 cm^−1^ (C=O stretching of the ester group), 1450 cm^−1^ (CH_2_ bending), 1180–1080 cm^−1^ (C–O–C stretching), and 870 cm^−1^ (CH bending of the amorphous phase) [34,35,36]. These bands appeared in both neat and modified PLA samples, with only minor variations in intensity after UP incorporation.

According to literature, UP resins exhibited characteristic peaks at 1690–1710 cm^−1^ (C=O stretching of carboxylic and ester groups), 1270–1250 cm^−1^ (C–O stretching), and a broad band around 3400 cm^−1^ corresponding to hydroxyl (O–H) groups [13]. In the PLA–UP blends, these functional groups overlapped with the PLA matrix bands, producing slight broadening and intensity changes in the C=O and O–H regions, particularly in PLA (2003D)–UP (3 phr), which suggests weak hydrogen bonding and secondary interactions between the resin and the polymer chains [16,37]. Moreover, at around 1500 cm^−1^ a peak attributed to the presence of the unsaturated UP resin structure was observed, corresponding to aromatic rings or conjugated double-bond (C=C) within its backbone. The slight variation in bandwidth became more pronounced following the modification of PLA 2003D.

Importantly, no new absorption peaks or significant frequency shifts were detected after UP addition, confirming that no chemical reaction occurred between PLA and UP during melt processing. The interaction between both components is therefore predominantly physical, governed by secondary forces such as hydrogen bonding and dipole–dipole interactions rather than covalent bonding. This behavior aligns with the biomimetic concept of non-covalent interfacial compatibility, where natural resin-like additives interact softly with polymeric matrices, enabling property modulation without chemical modification [16,37].

### 3.3. Mechanical Performance of TPS/PVA Blends

**Figure 5:**
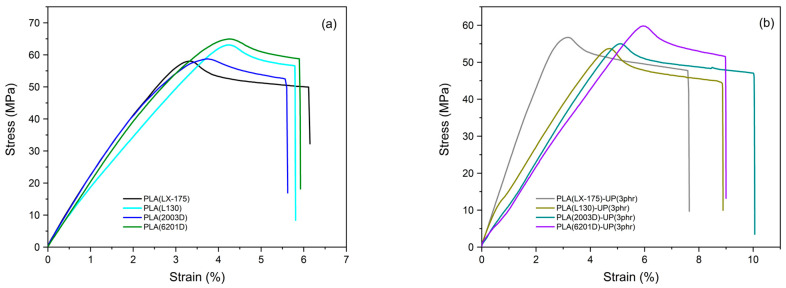
Stress–strain curves of PLA samples: (**a**) neat PLA grades; (**b**) PLA grades modified with 3 phr of UP.

As shown in Figure 5, the tensile behavior of the different PLA grades exhibited comparable trends in terms of Young’s modulus, elongation at break, and maximum tensile strength. The measured tensile strengths aligned with literature values for amorphous [38] and semi-crystalline [39] PLA. The amorphous matrices (PLA LX-175 and PLA 2003D) reached maximum strengths of approximately 58 MPa, while the semi-crystalline matrices (PLA L130 and PLA 6201D) exhibited higher values between 63 and 65 MPa—about 12% above those of the amorphous grades.

After incorporation of 3 phr UP resin, distinct changes were observed depending on the molecular weight and crystallinity of the PLA matrix (Figure 5b). The high-molecular-weight amorphous PLA (LX-175) displayed negligible variations in modulus and tensile strength but showed a slight 2% increase in elongation at break. In contrast, the low-molecular-weight amorphous PLA (2003D)-UP (3 phr) exhibited a 6.5% decrease in tensile strength and a 52% reduction in modulus, accompanied by an 80% improvement in elongation at break.

For the semi-crystalline grades, opposite effects were observed. The high-molecular-weight PLA (L130)-UP (3 phr) formulation showed a 52% increase in elongation at break but a 15% reduction in tensile strength, while the low-molecular-weight PLA (6201D)-UP (3 phr) presented decreases of approximately 8% in tensile strength and 42% in modulus, together with a 50% increase in elongation at break. These trends indicate that the UP resin acts as a low-molecular-weight plasticizing component whose influence depends on the molecular architecture of the PLA matrix.

As discussed by Thiyagu et al. [40], Comyn [41] and Fong et al. [42], the addition of plasticizers or small molecules enhances chain mobility by increasing free volume, thereby reducing intermolecular interactions. High-molecular-weight polymers, having longer chains and fewer chain ends, show less susceptibility to such mobility changes, whereas short chains exhibit greater free volume and segmental motion [43]. Accordingly, the increased chain mobility promoted by the UP resin resulted in a notable improvement in toughness for the amorphous, low-molecular-weight formulation (PLA 2003D-UP (3 phr)).

Comparable tendencies have been reported by Aldas et al. [44] who observed a decrease in modulus and an increase in elongation at break in starch/rosin-derivative blends, even at higher additive loadings (15 wt%). In PLA systems, amorphous regions are inherently more flexible than crystalline domains due to their disordered structure [44]. Thus, the moderate increase in elongation at break observed for semi-crystalline formulations can be attributed to the UP resin’s ability to hinder chain rearrangement, reduce crystallinity, and enhance deformability.

The toughness values, calculated from the area under the stress–strain curves (Table 4), confirmed these observations. All PLA–UP formulations exhibited increased toughness compared with their neat counterparts, attributed to enhanced chain mobility and localized energy dissipation enabled by the UP resin [45]. However, in Figure 5b a notable deviation is observed in the PLA (LX-175)-UP (3 phr) formulation compared to the other modified formulations, which is attributed to the reduction in Young’s modulus exhibited by the latter. In contrast, the apparent difference between PLA (LX-175) and PLA (LX-175)-UP (3 phr) arises from the significant enhancement in toughness (over 28%) and the slight increase in elongation at break (by approximately 2%), while maintaining a Young’s modulus comparable to that of the unmodified PLA (LX-175) formulation. This behavior suggests that the UP resin facilitates localized stress transfer and energy dissipation within the amorphous PLA network, leading to greater overall toughness without altering stiffness or inducing extensive plasticization. No strict correlation between toughness, molecular weight, and crystallinity could be established, as all formulations showed similar relative improvements consistent with their increased elongation at break, except for the amorphous, low-molecular-weight PLA (2003D)-UP (3 phr), which achieved the highest increase.

For comparison, Pawlak et al. [46] reported that maleinized linseed oil increased the toughness of semi-crystalline PLA (6201D) to around 3000 kJ·m^−3^, whereas the present PLA (6201D)-UP (3 phr) formulation reached approximately 3538 kJ·m^−3^, highlighting the superior reinforcing effect of the bio-based UP resin.

Overall, the results demonstrate that the incorporation of a natural, phenol-free rosin derivative effectively modulates PLA’s mechanical performance by altering molecular mobility and energy dissipation mechanisms. This plasticization-driven toughening behavior resembles biomimetic design principles, where hierarchical soft–hard interactions enhance flexibility without compromising overall material integrity.

### 3.4. Dynamic Rheological Analysis

Dynamic viscosity measurements were performed using a parallel-plate rheometer to evaluate the influence of UP resin on the complex viscosity of different PLA grades, considering the effects of molecular weight and crystallinity. The tests were conducted within the temperature range of 160–250 °C, following procedures reported in the literature [40]. As noted by Domenek et al. [45], understanding the viscoelastic behavior of PLA is crucial for assessing its processability and flow dynamics.

It should be noted that the melt flow index (MFI) values reported in Table 1 are reference data obtained under constant shear and load conditions (190 °C, 2.16 kg), whereas the complex viscosity measurements were performed under dynamic oscillatory shear. As these two tests probe different flow regimes—steady-state versus oscillatory—their results are not directly comparable. Consequently, similar complex viscosities between PLA LX-175 and PLA L130 can arise despite the differences in MFI reported by the suppliers.

**Figure 6:**
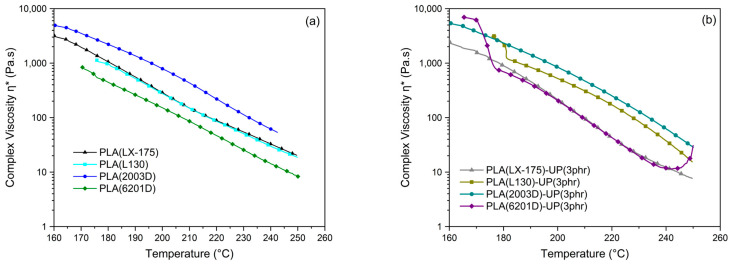
Evolution of the complex viscosity as a function of temperature for PLA samples: (**a**) neat PLA grades; (**b**) PLA grades modified with 3 phr of UP.

Figure 6 shows the variation in complex viscosity with temperature for the neat PLA grades and their corresponding PLA–UP formulations. As expected, viscosity decreased with increasing temperature, displaying the typical thermal thinning behavior of thermoplastic polymers, consistent with recent rheological studies highlighting the tunable flow response of PLA under varied processing conditions [6]. Among the PLA grades studied, the amorphous, low-molecular-weight PLA (2003D) exhibited the highest viscosity across the entire temperature range (Figure 6a). This finding contrasts with that observed by Garlotta [34], and Naser et al. [47], who reported higher viscosities for crystalline PLA grades, attributed to stronger intermolecular interactions and denser chain packing. The apparent discrepancy likely arises from differences in molecular weight among the commercial PLA grades used in this study, since molecular weight strongly influences melt viscosity. In most previous studies, amorphous and semi-crystalline PLA grades of comparable molecular weight were analyzed [28].

Upon addition of UP resin (softening point ≈ 135 °C), each PLA grade exhibited a distinct rheological response (Figure 6b). The lowest viscosity values were recorded for the formulation based on semi-crystalline, low-molecular-weight PLA (6201D-UP (3 phr)), particularly above 175 °C, where the polymer is fully molten according to DSC data. This suggests that the specific molecular architecture and entanglement density confer increased resistance to flow, or alternatively, that the unsaturated UP resin exhibits enhanced interactions with this structure, thereby leading to a higher viscosity at lower temperatures. Similar temperature-dependent rheological behavior of PLA has been reported in recent studies, where molecular rearrangements and additive interactions markedly influence complex viscosity [7]. For the amorphous, low-molecular-weight PLA (2003D-UP (3 phr)), UP incorporation caused no significant change in viscosity across the studied temperature range.

In contrast, semi-crystalline PLA grades exhibited an increase in viscosity after UP addition, particularly at lower temperatures (<190 °C). For the semi-crystalline, high-molecular-weight PLA (L130-UP (3 phr)), the viscosity enhancement persisted throughout almost the entire temperature range, whereas for PLA (6201D-UP (3 phr)), viscosity initially increased before declining beyond approximately 210 °C. This inflection may reflect a transition from restricted chain mobility to resin softening at elevated temperatures, increasing free volume and reducing intermolecular friction. Because UP is an amorphous, bio-based modified rosin resin, its partial miscibility and thermal softening can modulate polymer chain mobility and local flow resistance. In addition, the most striking difference is the sharp increase in complex viscosity (η* rise above 240 °C observed only for PLA (6201D-UP (3 phr)), which dictates the availability of reactive sites for the unsaturated UP resin. This behavior is a known rheological signature of chain-branching and cross-linking reactions in polymer melts. Therefore, Tin (II) 2-ethylhexanoate, which is used as a polymerization catalyst, could also act as a transesterification catalyst in the PLA melt, accelerating the thermal degradation of PLA by promoting chain scission and thereby generating more reactive end-groups (-OH and -COOH) at high temperatures. A higher concentration of these free end-groups acts as more initiation sites for the UP additive to start its cross-linking reaction, causing the faster viscosity spike [48,49].

A similar viscosity enhancement at low temperatures has been reported for PLA–rosin systems, attributed to transient physical interactions between the resin and polymer chains that modify the viscoelastic response [50]. From a processing perspective, the low-temperature range (<210 °C) is particularly relevant, as moderate viscosity increases can stabilize melt flow during extrusion, while higher temperatures may risk degradation or microstructural changes.

Overall, the incorporation of UP resin modulated the viscoelastic behavior of PLA in a manner dependent on molecular weight and crystalline structure. This adaptive flow response—where viscosity dynamically adjusts to temperature and molecular configuration—mirrors the biomimetic principle of functional adaptability seen in natural materials, which combine structural order with molecular mobility to achieve controlled deformation and flow.

### 3.5. Microstructural Evaluation

**Figure 7:**
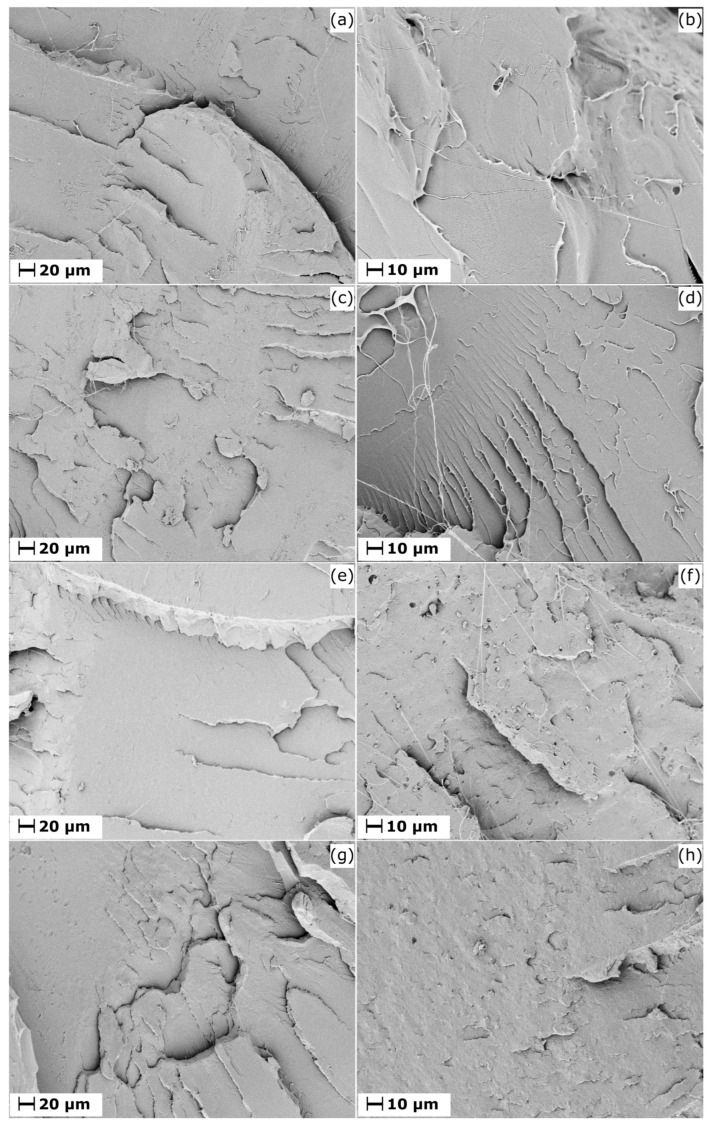
FESEM micrographs of the different PLA grades and their corresponding UP-modified formulations, obtained from the fractured surfaces of the impact test specimens: (**a**) PLA (LX-175) at 500×; (**b**) PLA (LX-175)-UP (3 phr) at 1000×; (**c**) PLA (L130) at 500×; (**d**) PLA (L130)-UP (3 phr) at 1000×; (**e**) PLA (2003D) at 500×; (**f**) PLA (2003D)-UP (3 phr) at 1000×; (**g**) PLA (6201D) at 500×; and (**h**) PLA (6201D)-UP (3 phr) at 1000×.

Field emission scanning electron microscopy (FESEM) was used to examine the microstructural features of the different PLA grades and their respective formulations with UP resin. Representative micrographs at 500× and 1000× magnification are shown in Figure 7.

Slight morphological variations were observed between the neat and modified PLA samples. The neat PLA matrices (Figure 7a,c,e,g) exhibited relatively rough fracture surfaces with grooves and layered features typical of brittle failure. In contrast, the PLA–UP formulations (Figure 7b,d,f,h) revealed finer fibrillar threads of torn material and small dispersed microdomains, likely corresponding to UP resin regions, along with occasional surface slits.

The presence of fibrillar threads indicates a more ductile fracture process, consistent with the increased elongation and toughness observed in the mechanical analysis. These fibrils suggest local plastic deformation facilitated by the UP resin, which enhances energy absorption during fracture. The small microdomains are attributed to UP resin particles not fully integrated into the PLA matrix during melt compounding. As Banerjee and Ray [18] noted, the morphology of polymer blends depends strongly on the intrinsic characteristics of the components and on the applied processing conditions. Hence, the limited dispersion of the resin may result from the specific extrusion parameters used.

Resin saturation can be ruled out, as previous studies have shown that concentrations below 3 phr do not reach a saturation threshold in PLA matrices [3]. The microdomains observed in PLA (2003D)-UP (3 phr) and PLA (6201D)-UP (3 phr) (Figure 7f,h) may also serve as initiation sites for cavitation, a mechanism that facilitates energy dissipation and contributes to the observed toughness enhancement [51]. This interpretation is supported by the correlation between the presence of microdomains, the ductile fracture morphology, and the improved mechanical performance of these formulations.

From a biomimetic perspective, the microstructural response of the PLA–UP systems reflect natural toughening mechanisms found in biological composites, where dispersed soft domains and fibrillar bridges promote controlled energy dissipation under stress. Such hierarchical deformation pathways illustrate how the integration of a natural, resin-derived component can impart adaptive, energy-absorbing behavior to a bio-based polymer matrix.

## 4. Conclusions

The incorporation of a phenol-free modified rosin resin (UP) into different PLA matrices produced distinct effects depending on the polymer’s molecular weight and structural organization. Thermogravimetric analysis confirmed that the thermal degradation of PLA remained essentially unchanged after UP addition, indicating that the resin does not compromise the inherent thermal stability of the polymer.

Conversely, DSC analysis revealed that UP resin significantly affected the thermal transitions of PLA. In amorphous PLA grades, both endothermic and exothermic events were suppressed, while in semi-crystalline, low-molecular-weight PLA grades, the cold-crystallization process shifted to lower temperatures. These observations demonstrate that molecular weight and crystallinity critically influence the interaction between PLA and UP, modulating the degree and kinetics of crystallization.

Mechanical testing showed that all PLA–UP formulations exhibited enhanced toughness, with increases above 25% in most cases and exceeding 60% for the amorphous, low-molecular-weight PLA. Rheological analysis further indicated that UP resin slightly increased viscosity in semi-crystalline grades, suggesting restricted chain mobility and temperature-dependent interfacial interactions.

Overall, the UP resin acted as a sustainable physical modifier that improved ductility and energy dissipation without altering PLA degradation stability. The most pronounced effects were achieved in amorphous, low-molecular-weight matrices, where the resin effectively promoted a biomimetic balance between flexibility and strength.

These findings highlight the potential of rosin-derived additives as bioinspired molecular modifiers capable of tuning the structure–property relationships of PLA-based materials. The approach demonstrates a pathway toward designing adaptive, eco-efficient polymer systems suitable for next-generation manufacturing applications, including biodegradable packaging, flexible films, and potentially, bio fabrication feedstocks where controlled viscoelastic and mechanical performance are critical.

## Figures and Tables

**Table 1:** Main characteristics and physical properties of the different PLA grades. The information was obtained from the suppliers’ technical data sheets and literature sources.

Physical Property	Commercial Name			
	Luminy^®^ LX-175	Luminy^®^ L130	Ingeo™ 2003D	Ingeo™ 6201D
Molecular weight (kg mol^−1^)	245 ^b^	170 ^b^	120 ^b^	59 ^b^
Density (g cm^−3^)	1.24 ^a,b^	1.24 ^b^	1.24 ^a,b^	1.24 ^b^
Melting temperature *T_m_* DSC (°C)	155 ^a^	175 ^a^	145–160 ^a^	155–170 ^a^
Glass transition temperature *T_g_* DSC (°C)	60 ^a^	60 ^a^	55–60 ^a^	55–60 ^a^
MFR (210 °C/2.16 kg) (g 10 min^−1^)	8 ^b^	23 ^a^	6 ^a,b^	15–30 ^b^
MFR (190 °C/2.16 kg) (g 10 min^−1^)	3 ^b^	10 ^b^	Not detailed	Not detailed
Stereochemical purity (% L-isomer)	96 ^a^	min. 99 ^a^	95.7 ^b^	98.6 ^b^

^a^ Provided by the supplier in the product technical data sheet. ^b^ Literature information [13,14].

**Table 2:** Composition and labeling of the different PLA grades and their corresponding formulations with UP resin.

Sample Label	PLA Grade	UP Content (phr)
PLA (LX-175)-UP (3 phr)	LX-175	3
PLA (L130)-UP (3 phr)	L130	3
PLA (2003D)-UP (3 phr)	2003D	3
PLA (6201D)-UP (3 phr)	6201D	3

**Table 3:** TGA and DSC thermal parameters of the different neat PLA grades and their corresponding formulations with UP resin.

	TGA			DSC					
Sample	*T_5%_* (°C)	*T_max_* (°C)	Char Residue (%) (at 600 °C)	*T_g_* (°C)	*T_cc_* (°C)	*ΔH_cc_* (Jg^−1^)	*T_m_* (°C)	*ΔH_m_* (Jg^−1^)	*X_c_* (%)
PLA (LX-175)	309.8	343.5	1.8	61.6	131.5	20.6	159.3	22.1	1.6
PLA (LX-175)-UP (3 phr)	304.7	341.7	1.6	61.4	132.7	8.7	158.8	8.7	<0.5 *
PLA (L130)	313.6	345.9	1.8	61.9	106.2	39.6	175.4	48.9	10.0
PLA (L130)-UP (3 phr)	310.4	345.8	1.3	62.1	109.3	34.4	174.8	46.4	12.9
PLA (2003D)	312.8	347.1	1.6	61.3	125.3	13.2	151.4	13.6	0.4
PLA (2003D)-UP (3 phr)	307.7	341.3	1.0	61.3	-	-	152.1	0.2	<0.5 *
PLA (6201D)	305.8	343.4	1.2	62.9	125.4	27.9	172.0	41.4	14.5
PLA (6201D)-UP (3 phr)	302.3	343.1	0.8	62.0	111.0	41.2	170.6	44.0	3.1

* Values < 0.5% correspond to negligible crystallinity within the experimental uncertainty of the DSC method.

**Table 4:** Comparative toughness values of the different PLA grades and their corresponding formulations with UP resin.

Unmodified PLA	Toughness (kJ/m^3^)	Modified PLA	Toughness (kJ/m^3^)	Toughness Increment (%)
PLA (LX-175)	2585.2 ± 30.4	PLA (LX-175)-UP(3 phr)	3313.9 ± 32.8	28.2
PLA (L130)	2411.5 ± 37.8	PLA (L130)-UP(3 phr)	3415.0 ± 41.5	41.6
PLA (2003D)	2372.9 ± 35.6	PLA (2003D)-UP(3 phr)	3939.5 ± 52.5	66.0
PLA (6201D)	2654.2 ± 46.7	PLA (6201D)-UP(3 phr)	3537.9 ± 37.2	33.3

## Data Availability

Data is available from the corresponding author upon reasonable request.

## Abbreviations

The following abbreviations are used in this manuscript:

PLA	Poly(lactic acid)
UP	Phenol-free modified rosin resin (Unik Print™ 3340)
TGA	Thermogravimetric Analysis
DTG	Derivative Thermogravimetry
DSC	Differential Scanning Calorimetry
*Tg*	Glass Transition Temperature
*Tm*	Melting Temperature
*Tcc*	Cold Crystallization Temperature
*T5%*	Onset Degradation Temperature (at 5% weight loss)
*Tmax*	Maximum Degradation Temperature
Δ*Hm*	Melting Enthalpy
Δ*Hcc*	Cold Crystallization Enthalpy
*Xc*	Degree of Crystallinity
FESEM	Field Emission Scanning Electron Microscopy
FTIR	Fourier Transform Infrared Spectroscopy
phr	Parts per Hundred of Resin (by weight)
Mw	Molecular Weight
L/D	Length-to-Diameter Ratio (of the extruder)
MFR	Melt Flow Rate
DMA	Dynamic Mechanical Analysis
UPR	Unsaturated Polyester Resin (general abbreviation, for comparison)

## References

[1] Nanda S., Patra B.R., Patel R., Bakos J., Dalai A.K. (2021). Innovations in applications and prospects of bioplastics and biopolymers: A review. Environ. Chem. Lett..

[2] Claro P.I.C., Neto A.R.S., Bibbo A.C.C., Mattoso L.H.C., Bastos M.S.R., Marconcini J.M. (2016). Biodegradable blends with potential use in packaging: A comparison of PLA/chitosan and PLA/cellulose acetate films. J. Polym. Environ..

[3] de la Rosa-Ramírez H., Aldas M., Ferri J.M., López-Martínez J., Samper M.D. (2020). Modification of poly(lactic acid) through the incorporation of gum rosin and gum rosin derivative: Mechanical performance and hydrophobicity. J. Appl. Polym. Sci..

[4] Thiruchelvi R., Das A., Sikdar E. (2021). Bioplastics as better alternative to petro plastic. Mater. Today Proc..

[5] Singhvi M.S., Zinjarde S.S., Gokhale D.V. (2019). Polylactic acid: Synthesis and biomedical applications. J. Appl. Microbiol..

[6] Rodríguez Hernández B., Lieske A. (2024). Widening the application range of PLA-based thermoplastic materials through the synthesis of PLA-polyether block copolymers: Thermal, tensile, and rheological properties. Macromol. Mater. Eng..

[7] Virág Á.D., Tóth C., Polyák P., Musioł M., Molnár K. (2024). Tailoring the mechanical and rheological properties of poly(lactic acid) by sterilizing UV-C irradiation. Int. J. Biol. Macromol..

[8] Arrieta M.P., Samper M.D., Jiménez-López M., Aldas M., López J. (2017). Combined effect of linseed oil and gum rosin as natural additives for PVC. Ind. Crops Prod..

[9] Karlberg A.T., Hagvall L. (2019). Colophony: Rosin in unmodified and modified form. Kanerva’s Occupational Dermatology.

[10] Ha Y.B., Jin M.Y., Oh S.S., Ryu D.H. (2012). Synthesis of an environmentally friendly phenol-free resin for printing ink. Bull. Korean Chem. Soc..

[11] Sun S., Weng Y., Zhang C. (2024). Recent advancements in bio-based plasticizers for polylactic acid (PLA): A review. Polym. Test..

[12] Pavon C., Aldas M., López-Martínez J., Ferrándiz S. (2020). New materials for 3D printing based on polycaprolactone with gum rosin and beeswax as additives. Polymers.

[13] Aldas M., Pavon C., López-Martínez J., Arrieta M.P.P. (2020). Pine resin derivatives as sustainable additives to improve the mechanical and thermal properties of injected moulded thermoplastic starch. Appl. Sci..

[14] de la Rosa-Ramírez H., Dominici F., Ferri J.M., Luzi F., Puglia D., Torre L., López-Martínez J., Samper M.D. (2023). Pentaerythritol and glycerol esters derived from gum rosin as bio-based additives for the improvement of processability and thermal stability of polylactic acid. J. Polym. Environ..

[15] Koçak E., Akkoyun Kurtlu M. (2024). Impact of production methods on properties of natural rosin added polylactic acid/sodium pentaborate and polylactic acid/calcium carbonate films. Int. J. Biol. Macromol..

[16] de la Rosa-Ramírez H., Aldas M., Ferri J.M., Pawlak F., López-Martínez J., Samper M.D. (2023). Control of biodegradability under composting conditions and physical performance of poly(lactic acid)-based materials modified with phenolic-free rosin resin. J. Polym. Environ..

[17] Plamadiala I., Croitoru C., Pop M.A., Roata I.C. (2025). Enhancing polylactic acid (PLA) performance: A review of additives in fused deposition modelling (FDM) filaments. Polymers.

[18] Banerjee R., Ray S.S. (2023). Role of rheology in morphology development and advanced processing of thermoplastic polymer materials: A review. ACS Omega.

[19] Dominici F., García D.G., Fombuena V., Luzi F., Puglia D., Torre L., Balart R. (2019). Bio-polyethylene-based composites reinforced with alkali and palmitoyl chloride-treated coffee silverskin. Molecules.

[20] Morris B.A. (2017). Rheology of polymer melts. The Science and Technology of Flexible Packaging.

[21] Bhasney S.M., Patwa R., Kumar A., Katiyar V. (2017). Plasticizing effect of coconut oil on morphological, mechanical, thermal, rheological, barrier, and optical properties of poly(lactic acid): A promising candidate for food packaging. J. Appl. Polym. Sci..

[22] Puchalski M., Kwolek S., Szparaga G., Chrzanowski M., Krucinska I. (2017). Investigation of the influence of PLA molecular structure on the crystalline forms (α′ and α) and mechanical properties of wet spinning fibres. Polymers.

[23] (2012). Plásticos—Determinación de las Propiedades en Tracción—Parte 2: Condiciones de Ensayo Para Plásticos Para Moldeo y Extrusión.

[24] (2010). Plásticos—Determinación de las Propiedades al Impacto Charpy—Parte 1: Ensayo no Instrumentado.

[25] Palai B., Mohanty S., Nayak S.K. (2021). A comparison on biodegradation behaviour of polylactic acid (PLA)-based blown films by incorporating thermoplasticized starch (TPS) and poly(butylene succinate-co-adipate) (PBSA) biopolymer in soil. J. Polym. Environ..

[26] Signori F., Coltelli M.B., Bronco S. (2009). Thermal degradation of poly(lactic acid) (PLA) and poly(butylene adipate-co-terephthalate) (PBAT) and their blends upon melt processing. Polym. Degrad. Stab..

[27] Carrasco F., Pérez O.S., Maspoch M.L. (2021). Kinetics of the thermal degradation of poly(lactic acid) and polyamide bioblends. Polymers.

[28] Atalay S.E., Bezci B., Özdemir B., Göksu Y.A., Ghanbari A., Jalali A., Nofar M. (2021). Thermal and environmentally induced degradation behaviors of amorphous and semicrystalline PLAs through rheological analysis. J. Polym. Environ..

[29] Wan Ishak W.H., Rosli N.A., Ahmad I. (2020). Influence of amorphous cellulose on mechanical, thermal, and hydrolytic degradation of poly(lactic acid) biocomposites. Sci. Rep..

[30] Pölöskei K., Csézi G., Hajba S., Tábi T. (2020). Investigation of the thermoformability of various D-lactide content poly(lactic acid) films by ball burst test. Polym. Eng. Sci..

[31] Chauliac D., Pullammanappallil P.C., Ingram L.O., Shanmugam K.T. (2020). A combined thermochemical and microbial process for recycling polylactic acid polymer to optically pure L-lactic acid for reuse. J. Polym. Environ..

[32] Piekarska K., Piorkowska E., Bojda J. (2017). The influence of matrix crystallinity, filler grain size and modification on properties of PLA/calcium carbonate composites. Polym. Test..

[33] Perinović Jozić S., Jozić D., Jakić J., Andričić B. (2020). Preparation and characterization of PLA composites with modified magnesium hydroxide obtained from seawater. J. Therm. Anal. Calorim..

[34] Garlotta D. (2001). A literature review of poly(lactic acid). J. Polym. Environ..

[35] Kumar S., Gupta S.K. (2013). Rosin: A naturally derived excipient in drug delivery systems. Polim. Med..

[36] Nair L.S., Laurencin C.T. (2007). Biodegradable polymers as biomaterials. Prog. Polym. Sci..

[37] Martín-Ramos P., Fernández-Coppel I.A., Ruíz-Potosme N.M., Martín-Gil J. (2018). Potential of ATR-FTIR spectroscopy for the classification of natural resins. Biol. Eng. Med. Sci. Rep..

[38] Zaaba N.F., Jaafar M., Ismail H. (2021). Tensile and morphological properties of nanocrystalline cellulose and nanofibrillated cellulose reinforced PLA bionanocomposites: A review. Polym. Eng. Sci..

[39] Nofar M., Mohammadi M., Carreau P.J. (2020). Effect of TPU hard segment content on the rheological and mechanical properties of PLA/TPU blends. J. Appl. Polym. Sci..

[40] Wilson R., George S.C., Anil Kumar S., Thomas S. (2017). Liquid transport characteristics in polymeric systems. Transport Properties of Polymeric Membranes.

[41] Comyn J. (2021). What are adhesives and sealants and how do they work?. Adhesive Bonding: Science, Technology and Applications.

[42] Fong R.J., Robertson A., Mallon P.E., Thompson R.L. (2018). The impact of plasticizer and degree of hydrolysis on free volume of poly(vinyl alcohol) films. Polymers.

[43] Promnil S., Numpaisal P.O., Ruksakulpiwat Y. (2021). Effect of molecular weight on mechanical properties of electrospun poly(lactic acid) fibers for meniscus tissue engineering scaffold. Mater. Today Proc..

[44] Aldas M., Ferri J.M., López-Martínez J., Samper M.D., Arrieta M.P. (2019). Effect of pine resin derivatives on the structural, thermal, and mechanical properties of Mater-Bi type bioplastic. J. Appl. Polym. Sci..

[45] Domenek S., Fernandes-Nassar S., Ducruet V. (2018). Rheology, mechanical properties, and barrier properties of poly(lactic acid). Adv. Polym. Sci..

[46] Pawlak F., Aldas M., López-Martínez J., Samper M.D. (2019). Effect of different compatibilizers on injection-molded green fiber-reinforced polymers based on poly(lactic acid)-maleinized linseed oil system and sheep wool. Polymers.

[47] Naser A.Z., Deiab I., Darras B.M. (2021). Poly(lactic acid) (PLA) and polyhydroxyalkanoates (PHAs), green alternatives to petroleum-based plastics: A review. RSC Adv..

[48] Spasojevic P., Seslija S., Markovic M., Pantic O., Antic K., Spasojevic M. (2021). Optimization of reactive diluent for bio-based unsaturated polyester resin: A rheological and thermomechanical study. Polymers.

[49] Cisar J., Pummerova M., Drohsler P., Masar M., Sedlarik V. (2025). Changes in the thermal and structural properties of polylactide and its composites during a long-term degradation process. Polymers.

[50] Kaavessina M., Distantina S., Chafidz A., Utama A., Anggraeni V.M.P. (2018). Blends of low molecular weight poly(lactic acid) (PLA) with gondorukem (gum rosin). AIP Conf. Proc..

[51] Nagarajan V., Mohanty A.K., Misra M. (2016). Perspective on poly(lactic acid) (PLA)-based sustainable materials for durable applications: Focus on toughness and heat resistance. ACS Sustain. Chem. Eng..

